# From Silence to Speaking Up About Sexual Violence in Greece: Olympic Journeys in a Culture That Neglects Safety

**DOI:** 10.3389/fpsyg.2022.862450

**Published:** 2022-04-06

**Authors:** Stiliani “Ani” Chroni, Anna Kavoura

**Affiliations:** ^1^Department of Sport and Physical Education, Faculty of Social and Health Sciences, Inland Norway University of Applied Sciences, Elverum, Norway; ^2^School of Sport and Health Sciences, University of Brighton, Eastbourne, United Kingdom

**Keywords:** sexual violence, patriarchy, collectivism, sport, Greece

## Abstract

The present study scrutinizes the role of societal culture in cases of sexual violence in Greek sport, as presented in the media after a two times Olympic medalist of Greece fired up the “‘me too’ Movement” in the country. Specifically, data for this study consisted of 36 media articles (14 international in the English language and 22 national in the Greek language), reporting multiple cases of sexual abuse and harassment in Greek sport and were published between January 2021 and January 2022. We drew on the cultural praxis heuristic to explore how the cultural setting operates as an underlying factor in priming athletes for harassment and abuse and in oppressing them into not speaking up. Our thematic analysis of media data revealed two overarching themes, namely, *keeping the home intact* and *failed negotiations with power*. Based on these findings, we discuss how subtle manifestations of patriarchy and collectivism perpetuate sexualized violence in Greek sport as they promote a climate of silence, prevent safeguarding, maintain underreporting of sexual violence, and delayed the arrival of the #metoo. We conclude that under the current circumstances, change seems to be a threat to all involved in Greek sport, yet for different reasons. For the coaches, sport officials, stakeholders, state system, change would require them to relinquish male powers and authority, find new meaning of what it means to be and do as a man, and allow women to be seen as counterparts. For the female athletes-survivors, it would require them to prioritize the self and their self-care and let go of the in-group loyalty and subordination learned and exhibited from infancy. We also contend that mere translations of international and regional safeguarding guidelines and toolkits cannot foster awareness raising, nor the implementation of measures within cultural settings that divert from the Global North. If we care to combat the universal phenomenon of sexualized violence in sport, a glocal approach is needed, where local socio-cultural factors are acknowledged, their role is addressed, and violence is understood within its context.

## Introduction

While sexual violence is a universal phenomenon, safe sport and safeguarding athletes are not. Though forms of sexual violence in sport (like harassment and abuse) drew attention in the end of 1980s (Brackenridge, 2001), today the magnitude of sexual (and other forms of) violence in sport remains understudied and a taboo (Parent and Fortier, 2017; Parent and Vaillancourt-Morel, 2021). According to the World Health Organization’s report on violence and health, sexual violence is about “any sexual act, attempt to obtain a sexual act, unwanted sexual comments or advances, or acts to traffic, or otherwise directed against a person’s sexuality using coercion, by any person regardless of their relationship to the victim, in any setting” (Krug et al., 2002, p. 2). With regard to forms of sexual violence, the IOC Consensus Statement (Mountjoy et al., 2016) defines sexual abuse as “any conduct of a sexual nature, whether non-contact, contact or penetrative, where consent is coerced/manipulated or is not or cannot be given” (p. 3) and sexual harassment as “any unwanted and unwelcome conduct of a sexual nature, whether verbal, non-verbal or physical” (p. 3). Studies with large samples conducted in Belgium, Canada, Netherlands, and United Kingdom evidence the prevalence and significance of the sexual violence problem (Alexander et al., 2011; Vertommen et al., 2016; Kerr et al., 2019), while studies conducted in countries outside Northern Europe and North America, like Israel, Czech Republic, Turkey, Greece, Japan, and Nigeria, to name but a few, further manifest the magnitude and highlight the globality of the problem (e.g., Fejgin and Hanegby, 2001; Fasting and Knorre, 2005; Gündüz et al., 2007; Chroni and Fasting, 2009; Takado et al., 2010; Elendu and Umeakuka, 2011). Cases involving multiple victims like Larry Nassar’s in the United States (Kerr and Stirling, 2019; Mountjoy, 2019), Bertrand Charest’s in Canada (Leavitt, 2017), or Fernando de Carvalho Lopes’ in Brazil (Reuters, 2018) bring to light how problematic sexual violence can be for the athlete as a person and a performer (Kerr and Stirling, 2019). In addition, the fact that in many cases people other than the victim and perpetrator “have known or suspected that athletes were being harmed” (Kerr and Stirling, 2019, p. 367), exposes the concealing that occurs within walls of silence that “shield and shelter” exploitative behaviors in sport and allow for perpetuation of abuse over time and across borders.

Both the magnitude and impact of sexual violence in sport, underline the importance of combating it and safeguarding athletes (Parent and Fortier, 2017; Kerr and Stirling, 2019) to prevent harm and ensure athlete safety and human rights. Global and regional recommendations and guidelines have been prompting nations and sport communities to create safe sport environments and establish safeguarding policies, strategies, and practices (e.g., International Olympic Committee Consensus Statement on Harassment and Abuse, Mountjoy et al., 2016; Conclusions of the Council and of the Representatives of the Governments of the Member States meeting within the Council on safeguarding children in sport, European Union, 2019). Today, there are still environments for which we lack knowledge about the implementation and effectiveness of any provisions for safeguarding. The state of Greece, which is the focus of this paper, is one of these. While we know of risks in sport associated with sexual violence (Marks et al., 2012; Parent and Vaillancourt-Morel, 2021), we have not adequately explored socio-cultural elements that may influence the ways of being and doing of athletes, parents, coaches, sport officials, and sport policy makers; and possibly hinder the implementation of safe sport policies and practices in certain societies and sport communities. According to Mountjoy et al. (2016), harassment and abuse are prominent in cultural contexts characterized by discrimination that is “based on power differentials across a range of social and personal factors” (p. 1). Expanding our understanding of how social factors can shape an environment, Skille and Chroni (2018) found that the national and sport cultures mingle and shape unique cultural features within a sport milieu. In the present paper, we scrutinize how the culture of Greece might be operating as a condition that shapes a shelter and a shield “for” sexual violence in sport and oppresses athletes who have been subjected to sexual violence from speaking up.

Sexual harassment occurs in Greek sport based on findings reported by Chroni and Fasting (2009) and Fasting et al. (2011, 2014). Incidents of sexual abuse and harassment in sport have also been reported in the media over the years, but until recently, such cases failed to attract attention and spark public debate on the topic or to mobilize efforts for progressive change. For instance, in 2012, the case of a male basketball coach who during his career assaulted at least 36 his male youth players, to “grow Spartans” as he said, raised a short-lived uproar in the Greek society (Sarris, 2012), but nothing changed with regard to the state’s policies and practices for safer sport. Ten years later, it was Sofia Bekatorou, gold medalist in 2004 Athens Olympics and bronze in 2008 in Beijing, who inspired wide public attention when she spoke out about being sexually abused at the age of 21 by a member of her federation’s board.

Sofia Bekatorou fired up the Greek #metoo campaign, which quickly trickled down to the milieus of performing arts and academia. The “me too” Movement was founded in 2006 by Tarana Burke with the aim to generate resources, support, and pathways that were not in place and to make healing possible for victims of sexual violence (me too, n.d.). What started as local work, became a global movement in 2017, when “the #metoo hashtag went viral and woke up the world to the magnitude of the problem of sexual violence” (me too, n.d.). The delayed onset of the #metoo in Greece, like in other places, was a matter of timing. Sofia’s disclosure was characterized as well-timed and vital (Karagianni and Panagiotou, 2021) for a society that is gender unequal and systematically sits at the very bottom of the EU Gender Equality Index (European Institute for Gender Equality, 2021). The “me too” Movement is grounded in understanding and disrupting male-perpetrated sexual violence and gender-based violence. Inside sport, better understanding of social factors and the power differentials formed within different cultural contexts (Mountjoy et al., 2016) could enhance understanding of and cause disruption to sexual violence.

Among the social factors that characterize the Greek societal context, a common denominator preventing people from recognizing, labeling, speaking of, and responding to sexual violence may be the collectivist patriarchic society (Chroni et al., 2013; Tsiganou, 2021). Patriarchy is prevalent in Greek society and is supported by highly influential institutions, such as family and religion (Tsiganou, 2021). Patriarchy was defined by Hartmann (1976) “as a set of social relations which has a material base and in which there are hierarchical relations between men, and solidarity among them, which enable them to control women. Patriarchy is thus the system of male oppression of women” (p. 138). Patriarchal beliefs of male, heterosexual dominance, and the devaluation of girls and women appear to be at the very root of gender-based violence (Lolis, 2020; Tsiganou, 2021). Indeed, cross-cultural comparisons reveal that highly patriarchal cultural contexts have higher rates of violence against women, compared to contexts with lower levels of patriarchal values (e.g., Ozaki and Otis, 2016). In the Greek society, people are primed to comply with hierarchical gender power relations and gender inequalities are accepted, normalized, and tolerated (Chroni, 2021a; Tsiganou, 2021). In a recent study, Tsiganou (2021) explored social representations, beliefs, and stereotypes in social media, daily newspapers and in interviews with authority figures and other professionals in Greece whose work also pertains to the management or treatment of incidents of domestic violence against women (judges, lawyers, police officers, doctors, nurses, and social welfare professionals). The study confirmed the role of patriarchy in facilitating and tolerating authoritarian attitudes and behaviors of men toward women, who are constructed as the inferior gender. Tsiganou (2021) further reported that the acceptance and normalization of violence in everyday life and the adoption of traditional gender roles are the strongest predictors of domestic violence. Patriarchal gender stereotypes dictate people’s attitudes, behaviors, and practices (Tsiganou, 2021).

Accordingly, the oppression of women in Greece is deeply rooted in the collective patriarchal consciousness, as the individual actions of both men and women, and the nature of their intimate relationships are affected by patriarchal biases. Traditional patriarchal beliefs about women’s subordinate roles (within the family, household, and workplace) and how women should be treated (need to be cared for, and at times also disciplined) justify gender inequality and violence occurring inside Greek institutions; sport being one of them. In particular, patriarchy continues to shape the ways of being and doing of the men and women of Greece as traditional patriarchal beliefs are subtly hidden within family values and traditions, the teacher-centric educational approaches, or a national team’s code of conduct expecting athletes to behave with due decency and decorum toward the (usually male) representatives of the sport’s authorities. Tsiganou (2021) also made a point of how the post-feminist alternative milieus failed (and continue to fail) in disturbing the dominant, male-centered gender norms in Greece; instead of being disturbed, the patriarchal structures of the Greek society continue to silence, absorb, or integrate anything alternative that tries to bring change.

In addition to the patriarchal values and gender relations, Greek people are primed into collectivism. They are nurtured and integrated in the realm of the family (and extended family with grandparents, uncles, aunts, and cousins), which shapes some early lived experiences within a strong, cohesive in-group that protects its members in exchange for loyalty. While the society is slowly transiting from a collectivist to an individualistic one (Hofstede, 1980), traditional orientations and values are still present (Pouliasi and Verkuyten, 2011; Chroni et al., 2013). It appears as if the society continues to hold on to the longstanding traditionalist axiom, “Fatherland, Religion, and Family,” fermented in the 1880s within Christian Orthodox groups aiming to confront the challenges of modernity, which brought in new ideas like feminism (Gazi, 2011). Feminism, as in promoting political, economic, personal, and social equality of the sexes, along with respect for women’s experiences, identities, knowledge, and strengths, was (and remains) contradictory to Greece’s implicit social rules and norms.

The shaping of early life compelling experiences with strong in-groups that protect their members in exchange for loyalty, can facilitate the dominant patriarchal, male-centered Greek structures to silence, absorb or integrate anything alternative, and thus remain intact. People are nurtured into abstaining from sharing in-group facts with those not belonging to the group (τα εν *o*íκω, μη εν δήμω translating as what happens at home, stays at home); defying the rule of in-group solidarity by revealing facts harmful to the group is contemptible. Sport is a strong, male-dominated, cohesive in-group, and according to Kerr et al. (2020), relationships in sport often resemble those in a traditional family. While the closeness, commitment, complementarity, and co-orientation qualities are desirable as they form strong relationships, they can also be risk factors as they increase athlete vulnerability within the power differential present in sport (Kerr et al., 2020). Power imbalance and misuse of a position of power appear to lie beneath all forms of maltreatment (Fasting and Brackenridge, 2009; Stirling and Kerr, 2009). In light of this and the Greek sport context, an abused athlete’s silence, and a sport organization’s concealing of violence are indications of solidarity, loyalty to the in-group of sport and submission to hierarchical power structures.

Researchers have considered several risk factors for sexual abuse in relation to the sport environment, but few studies have provided evidence for these (Parent and Vaillancourt-Morel, 2021). Risk factors relating to individual vulnerabilities (e.g., being woman and younger, athlete with low self-esteem, absence of parent) as well as to the sport and situational ones (e.g., access to young people, private training sessions, and traveling) were identified early on by Brackenridge (1997, 2001). Vertommen et al. (2016) found that ethnic minority, sexual minority (LGB), athletes with disabilities, as well as athletes competing at the international level were overrepresented among survivors of interpersonal violence, in a sample of 4000 adult athletes from the Netherlands and Belgium who were surveyed about their childhood experiences in sport. Furthermore, the Dutch and Belgian women reported a higher prevalence rate of sexual violence, the men reported more experiences of physical violence, while there was no significant gender differences in the prevalence of psychological violence. According to Parent and Vaillancourt-Morel (2021), in their study with 1055 Canadian youth athletes, sexual preference and sport level were found to be associated with experiences of sexual violence, which is in agreement with the findings of Vertommen et al. (2016). With reference to abusive coach–athlete relationships, Kerr et al. (2020) suggest that closeness in the relationship, power, authority, trust, and the deference afforded to coaches require our attention. Malina (2010) had suggested, along similar lines, that early sport specialization may be another risk factor as it increases the young athletes’ vulnerability to abuse by those in positions of authority.

As articulated in the Start to Talk initiative of the Council of Europe (n.d.) website:

*Sexual violence thrives when there is tolerance for discrimination, physical violence and inappropriate sexual behavior. Imbalanced power relations, authoritarian leadership and rewards structures create relationships based on fear and dependence and hence vulnerability to the abuse of power. The loss of “natural barriers” because of the physical contact required and the sharing of showers, changing rooms and confined spaces with adults also expose children to several forms of sexual violence. And of course, there is the scandal avoidance by organisations and individuals who prefer to hide the abuse, thus sacrificing the victim and giving the abuser a licence to harm. (Sexual abuse happens in sport too, Start to Talk, https://www.coe.int/en/web/sport/start-to-talk)*

The people of Greece appear to be beyond tolerant to gender discrimination as exemplified by the country’s last place in the EU Gender Equality Index (European Institute for Gender Equality, 2021). Discrimination, sexism, the devaluation of women in everyday life and the media, the rape culture, the defying of victim credibility, the victim-blaming and shaming are normalized practices (Tsiganou, 2021), rarely addressed before Bekatorou’s disclosure.

Overall, in countries like Greece where loyalty to cultural silence is the norm, the attention given to sexualized violence in sport continues to be reactive, sparse, and futile. Understanding the socio-cultural conditions, the loyalty and the culture of silence can shed light on why victims do not speak up, and sexual violence is not interrupted, and also identify areas in which we could intervene to mobilize change. The research question we wished to answer with the present study is: What may be the underlying socio-cultural factors that prime female athletes (and the sport environment) of Greece toward accepting, tolerating, silencing, and concealing sexual abuse occurring inside sport? In what follows, we engage the cultural praxis heuristic (Ryba and Wright, 2005; Chroni and Kavoura, 2020) and perform media analysis to look at cases of sexual violence experienced by Greek female athletes.

## Methodology: Materials and Methods

### Theoretical Framework

To explore socio-cultural factors related to sexual violence in Greek sport, we drew upon the cultural praxis heuristic proposed by Ryba and Wright (2005). We strategically utilized the cultural praxis framework to explore how the cultural setting of the country operates as an underlying factor in priming athletes to harassment and abuse and in oppressing them from speaking up. Cultural praxis is a critical approach to sport psychology that uses cultural studies to highlight the complex interactions of power that are sometimes taken-for-granted in sport and the sport sciences (Chroni and Kavoura, 2020). Specifically, this heuristic is asking us to step away from singular understandings of sport, and instead consider the phenomena that we are studying through a social justice and culture-sensitive lens (Ryba and Wright, 2010). It calls for blending theory, research, and practice (see Ryba, 2017) in ways that enhance our understandings of how to provide wholistic support and structures of care to athletes of all backgrounds and identities. Previous cultural praxis scholarship helped us to unravel the socio-cultural characteristics of the Greek sporting context that might constrain the experiences of Greek athletes (see Chroni et al., 2013; Kavoura et al., 2015) as applications of the cultural praxis framework can be found in the content areas of athletes’ careers and in understanding minority athletes’ and coaches’ worldviews, experiences, and identities (see Chroni and Kavoura, 2020 for an overview of existing research). To our knowledge, the potential of the cultural praxis heuristic has not yet been fully realized in the content area of sexual abuse in sport, even though several studies have emphasized the role of the cultural context in the perpetuation of these troubling phenomena (e.g., Brackenridge, 1997; Fisher and Anders, 2019; McMahon et al., 2020). In this paper, we use cultural praxis as the “how to” of conducting culturally sensitive, localized, and reflective research on the understudied topic of sexual violence in Greek sport.

### Data

In countries where we still lack data on sexual violence in sport and research on this topic has been met with resistance, scholars suggest that the use of secondary sources of data. Sources like media and court reports, police files, and data from criminal registers (Helweg-Larsen and Larsen, 2005; Brackenridge et al., 2008; Fasting et al., 2013; Sanderson and Weathers, 2020) can offer valuable information and help us understand sexual violence in sport. Accordingly, the present study draws on national and international media articles that reported cases of sexual abuse and harassment in Greek sport and were published between January 2021 and January 2022.

To identify the articles, we searched the European Newsstream database, as well as Google, using different combinations of the words, “sexual abuse,” “#metoo,” “sport,” “athlete,” “Sofia Bekatorou,” and “Greece” (in Greek language, “σεξ*o*υαλική κακ*o*π*o*íηση,” “#metoo,” “αθλητ*,” “Σ*o*φíα Mπεκατώρ*o*υ,” and “Eλλάδα”). These key terms were selected as they reflect the phenomena (sexual abuse), study population (athletes), and context (sport) that were of interest to this study. The term #metoo was also selected as it is a popular hashtag that athletes use when reporting experiences of sexual abuse in the media. We also decided to use the name “Sofia Bekatorou” as a search term, as in addition to firing up the #metoo movement in Greece, she has become a key figure in advocacy work related to sexual abuse and her name is often mentioned in the media in relation to other athlete-survivor stories.

In our dataset, we included media articles that included direct quotes from athletes-survivors to ensure that the athletes’ perspectives were represented in the data and excluded media articles in which the focus was not on the sport context, even if there were brief mentions to it. For example, articles that reported cases of sexual abuse in the context of arts. We also excluded duplicates and articles that focused on other national contexts, such as Cyprus. However, we decided to include articles in which the focus was physical and emotional abuse in sport, as these also relate to the culture of silence and facilitate sexual abuse. In total, this study is based on 36 articles that met our criteria; 14 international articles in the English language and 22 national articles in the Greek language. When presenting quotes from the Greek articles in our findings and discussion, these have been translated into English. While more articles can be found in the media, we stopped searching for data when our analysis reached saturation of codes (Saunders et al., 2018).

### Data Analysis

To uncover patterns in the data, we followed a social constructionist approach to thematic analysis, as described by Braun et al. (2016). Thematic analysis, as Braun et al. (2016) pointed out, is a versatile method for a range of research purposes, including determining how a topic of interest is formed in the media. As a first step, we engaged with the data at a semantic level to identify challenges, barriers in facilitating violence and in oppressing the speaking out about violence. Second, we coded the data, following a latent approach focused on the underlying cultural mechanisms that allow for abuse to happen and raise walls of silence in the Greek sport context. The codes were then developed and arranged into themes (Braun and Clarke, 2019), which we continued to refine and edit, looking also for disconfirming patterns that contradicted our evolving interpretations. While the coding of the raw data was data-driven, the development of the themes and overarching themes was both data- and theory-driven as existing knowledge helped us make better sense of the codes identified, and organize these in the most meaningful way (an approach also used by Skille and Chroni, 2018). This work of organizing data-driven codes and making meaning was informed by the cultural praxis framework’s notion of multiplicity regarding voices of cases and our interpretations, as well as by previous scholarship on sexual violence in sport, and literature on gender-based violence and gender stereotypes in Greece. During the whole process, we had extensive discussions about the politics of research on such a highly sensitive topic and we were conscious that themes did not simply “emerge” from the data but were generated through the collaborative efforts of the two researchers (Braun and Clarke, 2019). While we wanted to make sure that athletes’ voices are represented in the data, our focus on the underlying cultural context led us to include in our analysis also other voices, such as those of the journalists, perpetrators, lawyers, politicians, and social scientists that were quoted in the media data.

### Researchers’ Reflexivity and Ethical Considerations

When carrying out reflexive cultural sport psychology research, scholars must acknowledge their own experiences and subjectivities, as well as their influence in the study process (McGannon and Johnson, 2009; Ryba, 2009; Schinke et al., 2012). Thus, our reflective account is presented here focusing on how our subjectivities, past experiences in sport, and epistemological situatedness played a role in conceptualizing the study, reviewing the literature, making sense of and interpreting the media data, and developing this manuscript.

We are Greek women, feminist and cultural sport psychology researchers, who have left Greece in order to pursue transnational academic careers abroad. Yet, we see ourselves as insiders in the world of Greek sport, as we both have trained, competed in, and coached (different sports) in Greece for several years. Through our advocacy and scholarly work, we have tried to mobilize change for all, and women in particular and bring something back to the sports that we loved and their communities (e.g., Chroni and Fasting, 2009; Kavoura et al., 2015). Having lived, trained, researched, and provided applied sport psychology services in Greece, we felt equipped to understand the cultural complexities of the Greek society, sport system and the difficulties women face when trying to navigate in this male dominated terrain. Particularly, our experiences in the Greek sport settings echo the literature findings of women who feel discomfort with the dominant patriarchal understandings and the unequal gender power relations that are engrained in our sporting contexts (Kavoura et al., 2015). When thinking of our training practices and work in Greece, we can both recall times during which we felt gender harassed (discriminated because of our gender), sexually harassed (in the form of unwanted sexual attention and comments; see Chroni and Fasting, 2009), and sexually abused (in the form of unwanted sexual coercion, see Chroni and Kavoura, 2022). Moreover, as insiders to the Greek sport culture, we recognize that we share the same understanding of social practices and our subjectivities have also been constructed through negotiations with the same cultural discourses (see about negotiation processes of women sport scientists, Chroni et al., 2021). However, as gender scholars, living and working in contexts that are considered to be more egalitarian and safer, we were also able to distance ourselves from the taken-for-granted cultural understandings and interrogate the dominant discourses and socio-cultural practices that exist in our home-culture and might be responsible for the walls of silence that exist around sexual violence in sport.

Given the sensitivity of this research topic and pre-existing relations with survivor-athletes whose stories have been depicted in the media and were used in this study, our research was guided by relational ethics (Ellis, 2007). According to Ellis (2007), relational ethics prioritize ethics of care (Gilligan, 1982), mutual respect, dignity, and connectedness, between the researchers, the researched as well as the communities and the settings they study. Drawing on the principles of relational ethics, we were guided on moral, ethical, and methodological level by the question “What should [we] do now?” (Ellis, 2007, p. 4). We acknowledge here that the first author had extensive ongoing conversations with one of the athletes-survivors and that we both were more familiar with one of the stories and not all of them. While the content of the conversations was not used in this manuscript, it triggered discussions between us regarding how we would approach the stories “in a humane, non-exploitative way, while being mindful of our role as researchers” (Ellis, 2007, p. 5). We wanted to be “caring, fair, and transparent” to all the women who would help us learn through their words, regardless of knowing them in person or not, and avoid being influenced by some stories more than others. We wanted to treat the women, their stories and ourselves with impartiality and respect, to tell the story of a hurtful Greek culture without violating the intensity of the lived experiences, but also, we wanted to protect and preserve existing relationships. The richness of the data and parallels in these women’s stories helped us focus on the content and on uncovering socio-cultural factors that conditioned them into accepting violence and not speaking up. Upon completing the analysis and looking back at how we treated the data, what we identified within it, we believe that what conversations with athletes-survivors did was to inspire and commit us to continue working through the ugliness of sexual violence stories and translate these into knowledge that can bring change in our native country.

## Findings and Discussion

The media articles that we analyzed, reported nine cases of sexual violence, in the sport contexts of athletics, gymnastics, sailing, basketball, water polo, and swimming. Perpetrators were all males in positions of power, such as federation officials, coaches, sport medics or fellow elite athletes. The victims were underage or young adult female elite athletes. Behaviors included sexual harassment in the form of unwanted sexual comments, unwanted touching and forcing the athlete to strip, as well as more aggravated forms of sexual abuse including rape (for definitions of terms see Krug et al., 2002; Mountjoy et al., 2016). One of the cases, involved multiple victims and perpetrators and pertained to sexual, physical and/or psychological abuse that was prolonged over time. In exploring socio-cultural elements that might *shelter* sexual violence over female athletes and could help us understand how the walls of silence that surround sexual violence in Greek sport are built, maintained, and keep out safeguarding, our thematic analytic procedure revealed two overarching themes: (1) *keeping the home intact* with two subthemes, (a) holding back on change and (b) staying with the known; and (2) *failed negotiations with power* also with two subthemes, (a) negotiating with national and sport culture glitches and (b) preserving power. Some of the themes relate more to the collective dynamics of the environment, while other ones to individual negotiations that happen within this system. In what follows, we present and discuss the themes.

### Keeping the Home Intact

To *keep the home intact*, the collective side *via* its hereditary prowess and powers is *holding back on change* and progress, as the individual victim female-athlete is accepting the hierarchy and is *staying with the known* that feels comfortable. “Home” is not used literally here, and is a colloquialism for the larger milieu of Greek sport, sport stakeholders, politics, policy makers, sport organizations, but also to the victims, their homes, families, environments. Holding back on change is realized *via*, outdated, authoritarian, abusive coach-centered coaching, and training practices; a legal code and practices that do not recognize a victim’s trauma, involve non-supportive conditions, lack protective mechanisms, overlook the time it takes for a victim-survivor to come forward; and lastly policy change that does not materialize (see Table 1).

**TABLE 1:** Thematic map of overarching theme *keeping the home intact* illustrating socio-cultural elements that shelter sexual abuse of female athletes in Greece.

Codes	Themes	Overarching theme
Outdated, abusive coaching, training practices	Holding back on change	Keeping the home intact
Untrustworthy legal code, practices, missing mechanisms		
Broken promises for change		
Protecting the institution of family	Staying with the culture’s “known”	
Drawing on religion		

The Greek sport context was portrayed by some athletes as a dreadful culture that operates with outdated, rough coaching and training practices, where the coach exercises power wrongfully causing physical and psychological harm and where athlete-centered coaching that cares for athlete well-being and safety is not practiced. As an international article describes:

*… coaches would slap, kick, shove and throw objects at them during training, even dragging some girls by the hair and grabbing them by the crotch. On occasion, coaches would remove protective mats, causing injuries. Some of the athletes were forced to train while injured…. Disciplinary measures allegedly included forcing athletes to train in extreme temperatures and denying them toilet breaks. Because of strict weight requirements, some athletes starved themselves to the point of fainting, and resorted to secretly eating toothpaste and food leftovers scavenged from hotel bins… (Hadoulis, 2021)*

Based on developments in knowledge on athlete training and coaching, scholars proposed a shift toward athlete-centered coaching two decades ago (Lyle, 2002; Kidman and Lombardo, 2010). Athlete-centered coaching prioritizes and actively cares for athlete well-being (Campbell et al., 2021), takes a holistic approach to athlete development through ownership, responsibility, initiative, and awareness (Pill, 2018), and prevents athlete maltreatment (Kerr and Stirling, 2008). According to Gervis and Dunn (2004) and Kerr and Stirling (2008) unequal power dynamics between coaches and athletes and abusive coaching practices (emotional, physical, and sexual) are predisposing factors of sexual maltreatment. In addition, based on a study with 104 female athletes in Greece, authoritarian coaching behaviors is a strong predictor of sexual harassment experiences (Sand et al., 2011). The coach-centered approach and abusive coaching practices presented in the quotation above were reported years later after they happened indicating the priming of these athletes to accept this as the price to pay on the way to better performances (Chroni, 2015).

Within an unequal and abusive context like this, it is not surprising that athletes feel that speaking up is not safe and would not lead to justice, even years after the abuse was experienced. The legal system, its processes and enforcers discourage victims from speaking up and appear not to be trusted neither by the athletes-survivors, nor by their lawyers. As a lawyer dealing with cases of sexual abuse said in a Greek article, “…we must stop telling the victims to ‘talk’. ‘The victim will talk when the state really helps, both with welfare structures and with structures that suppress the perpetrators’” (Sayias, 2021). Chroni (2021b) characterized this call to athletes to speak up as premature, naïve, and a leap of faith, considering also how much is at stake for the athlete who risks losing the dream to excel once she breaks her loyalty toward her sport (see also Chroni, 2015). The system is outdated and is failing the athlete-survivors who seem to be punished anew when they cannot find justice after trying so hard to gather the courage and strength to speak up. As described in international articles, “No one will be criminally charged as the alleged offense took place too long ago” (Kottis, 2021); “Although the 20-year statute of limitations on rape has expired in her case, the government is considering extending it – though not retroactively” (Psaropoulos, 2021). No procedural change has been implemented in the 12 months since sexual violence in Greek sport took center-stage.

In a “home, a family” that is failing its “children,” building the courage to speak up can take time. Time is relevant and each victim-survivor heals in their own time; a fact difficult to grasp, for a public that is ignorant of the trauma caused by sexual violence and places blame on victimized women. As Sofia Bekatorou explains in an interview article:

*The classic question was ‘why did you speak now’ which was expressed by people who have no sense of what a trauma means and I do not misunderstand them or better yet, misunderstand those who do not say it meanly. What they need to learn is that trauma is timeless. You cannot experience it in the time that has been done. Each person needs a different amount of time to process it. One can react immediately, one can take 20 years, one never. But to react even a few minutes before one dies, is good for the person. One cannot turn around and say, ‘why now?’ Because now [the person] could, because now [the person] developed the defenses to face it. (Tsilochristou, 2021)*

Tsiganou (2021) in her research on how professionals tackle domestic violence in Greece, pointed out that judges’ interpretations of the law are influenced by dominant patriarchic stereotypes. Similarly, Lolis (2020) pointed out the high levels of patriarchal values in Greece, as male dominance and toxic masculinities are endorsed by the police. In the quotations below, a lawyer shares her view of a system that lacks protective mechanisms (Public Radio International, 2021), and Sofia Bekatorou elaborates on how the environment and conditions induce shame on a victim who dared to come forward (Sayias, 2021).

*Stentoumi [a lawyer] said she hopes attention on the issue will bring about changes in the process for reporting sexual abuse. She said the current system deters and traumatizes victims – as police officers are not adequately trained on these kinds of cases. Also, people who want to report rape and other types of abuse often have to wait days to be examined due to a national shortage of medical examiners (Public Radio International, 2021)*

*Violence has existed, exists and we hope it will not exist in the future. Too many victims now file complaints, not because there was no abuse and harassment before, but because the environment and conditions did not allow anyone to speak openly. The victim felt guilty (Sayias, 2021)*

*“Unfortunately, a lot of disgusting comments are disguised as humor. And you know as a female athlete that this makes you feel uncomfortable. You just don’t know how to put it into words or who to talk about this [with].” Glyniadaki was a minor when some of this was happening. She and her teammates would sometimes talk among themselves, but they didn’t feel they had anywhere to go for help. “We didn’t feel like we had the power or the allies to go forward and make this an issue.” […] In this environment, conversations that challenge institutions still dominated by men have been stalled. “This is a turning point for sure,” Glyniadaki said. But people who are feeling empowered to speak are also facing a backlash. “There’s … a lot of victim-blaming, a lot of slut-shaming. The environment around is very hostile for [women coming forward].” Diana Manesi, senior researcher with Diotima, a longstanding feminist nongovernmental organization in Greece, said. (Public Radio International, 2021)*

Humor or “disgusting comments … disguised as humor” that was mentioned above, has been found to be a masking tactic against gender-based violence (Cole, 2015; Lockyer and Savigny, 2020) but also a behavioral response of victims when attempting to confront their perpetrators (Fasting et al., 2007).

The past year reviewed here through media stories of sexually abused athletes in Greece, appears to have been filled with promises that did not translate into actions. “The Hellenic Olympic Committee and the government is urging other sex abuse victims to speak out” (Vassilopoulos, 2021) but reporting mechanisms that safeguard the athlete-survivor are still not in place. As Tiivas, the CEO of SafeSport International said in an international article, “whether the Greek ‘me too’ phenomenon Bekatorou triggered turns out to be a watershed moment or mere infotainment depends on how the government now tackles change” (Psaropoulos, 2021). While measures were announced, their implementation did not progress enough and as Stratigaki (2021) argued, the Greek government missed opportunities for legislative improvements in the area of combating gender-based violence. One of the measures announced by the Ministry of Sport in February 2021 was the development of codes of conduct by national sport federations. Whether codes of conduct were developed or not by the federations is yet to be monitored according to a recent interview by the Minister of Sport (Papadakis, 2022). Hence, the society, the system, the politicians, the law enforcers, the sportspeople appear to hold back on change and progress.

The *keeping the home intact* overarching theme was also grounded on individuals *staying with their culture’s known* that was built on two codes pertaining to how individuals attempt to protect the institution of family and how they fall back on the other strong institution of religion. Traditionally speaking, keeping a Greek home intact is a house-chore that was (and continues to be) within a woman’s caretaking gendered role (Costa, 2005). Here, while abused athletes feel the harm done onto them, they continue to protect the institution of family by keeping what they experienced quiet: “I kept it secret for many years. He was married with children and the reason I did not say so was because I respected his children and nothing else” (Agence France-Presse, 2021). “Rape in every sense. I was then 11 years old. I was in shock and instead of supporting me, they wanted to destroy me. It was very difficult. ‘I just did not want my family to go through it then’” said an athlete in her deposition (Naftermporiki, 2022). Perpetrators take advantage of this and construct themselves as “family men” whose children need to be protected. Adamopoulos upon being identified by Sofia Bekatorou as her abuser, called the authorities to help him safeguard his extended family, “I call on the state, institutions and the media to first respect my family, my children and grandchildren” (Vassilopoulos, 2021).

The societal pressure on the victim-survivor is immense and was addressed in a case presented by Chroni (2013a) where a parent confronted the football club’s president for inappropriate touching of young female players to which he responded, “What do you want now to happen? Ruin my family life?” This pressure to care for the abuser’s family, as shown in previous paragraph’s quotations aims to sensitize the victim-survivors on cultural values, which in Greece means showing respect (bowing) to the authorities along with showing solidarity and loyalty to the in-groups within which they were brought up, regardless of the costs. As such, when push comes to shove, care for the athlete is nowhere to be found. While international literature emphasizes holistic care for athlete well-being (Campbell et al., 2021), this is not the case for athletes in Greece.

When a victim’s physical family and sport-family do not provide the necessary protection to the athlete-survivor, either because she is protecting them by not speaking up or because she was disloyal and exposed her sport-family’s dirty laundry, some athletes turn for help to the other strong institution they know of, religion, “I was getting the medals and I could not be happy. For many years I said I had to speak [up]. Eventually I disappeared and went to a monastery for a short time and prayed, Devetzi said” (Agence France-Presse, 2021). However, the institution of Greek Orthodox religion is as patriarchic and collectivist as the society (Tsiganou, 2021), hence instead of peace of mind and healing, the abused athlete may find herself left shamed.

*Spyridoula Athanasopoulou-Kypriou, a feminist theologian in Athens, has been thinking about the role of the Greek Orthodox Church, a powerful and influential institution in Greece that is led exclusively by men, in all of this. ‘I’m adamant that it is because of the church that things do not progress, ‘ … While the church has publicly signed on to initiatives to combat gender-based violence, including domestic abuse, it ‘perpetuates inequality and violence against women’ through its teachings and actions. She added that church leaders and members often undermine people who come forward with abuse allegations by sympathizing with alleged perpetrators. “You have priests … who will tell you, ‘Poor guy, he was in a difficult situation. It was the pandemic. He has some problems.’” (Public Radio International, 2021)*

In sum, it appears that to keep the Greek sport home intact, the different ends (collective and individual) meet in cultural institutions and traditions, outdated premises and practices, and (un)conscious deafness and blindness, where they interact, reconstruct, and perpetuate cultural inequalities, discrimination, and abuse. The theme of keeping the home intact, denotes that the challenges of change are not confronted yet, and that power remains on the side that it was.

### Failed Negotiations With Power

As an overarching theme, *failed negotiations with power* is about individual *negotiations with glitches of Greece’s national and sport culture*, along with collective acts that may well *preserve power* for those who have it. As negotiations with problematic aspects of the national culture we regarded situations where the individual athlete negotiates between caring for her emotional vulnerabilities and remaining silent, as well as when negotiating between remaining silent and not protecting herself or others in her sport from maltreatment and abuse. Negotiations involving the sport culture, place the individual at a juncture where she must choose between her own safety and well-being, and her Olympic dream (see Table 2).

**TABLE 2:** Thematic map of overarching theme *failed negotiations with power* illustrating socio-cultural elements that shelter sexual abuse of female athletes in Greece.

Codes	Themes	Overarching theme
Emotional vulnerability vs. silence	Negotiating with glitches of national and sport cultures	Failed negotiations with power
Safety and well-being vs. the Olympic dream		
Silence vs. in-group solidarity		
It’s not about sex, it’s about power	Preserving power	
Corrupted management		

Being caught between one’s emotional vulnerabilities as an athlete, like adoring your sport and wanting to stay with it, and remaining silent while harming yourself, was a common experience in the athletes’ stories in the media. As an athlete said, “We have been saying this [being abused] for years …but they did not take us seriously because they know how much we love [the sport]” (Hadoulis, 2021). Another athlete talked of being systematically raped and taking her more than 10 years to realize that a child cannot be blamed for being raped,

*There was no consensus on my part. He threatened to kill me and destroy my family if I talked and revealed what he did to me, ‘the girl had said in her testimony.’ A year ago, I found the courage to denounce my systematic rape, the physical and verbal abuse I was subjected to by a man I trusted. It took me ten years to realize that a child cannot be to blame. (Samou, 2022)*

While the athlete-survivors of the study construct this negotiation as one they are responsible for, the literature suggests that victim shaming and blaming are common strategies used by perpetrators to weaken the targeted victim and to secure secrecy (Brackenridge, 2001; Sinnamon, 2017). The athlete-survivors’ quotes above illustrate how well these women have been primed, groomed in this culture of shaming and blaming that asks victims to take responsibility for the harm done onto them.

The negotiation between self-care in the form of speaking up and seeking justice and continuing with one’s elite sport dreams is a reality that researchers have pointed out for years (e.g., see Brackenridge, 1997, 2001; Chroni, 2015). As illustrated by Chroni and Kavoura (2022), Bekatorou could not speak up when losing the sport she loved and the Olympic dream were at stake. The cost of exposing the in-group of sport is high, and for one of our athletes it was the end of her career, “high-performance sport stopped the day I decided to talk about my rape. I knew my career would end somewhere there, not because I spoke up, but because I realized that it was important to give a different fight” (Tsilochristou, 2021). Greek athletes are afraid to talk, and “she had kept quiet over the alleged abuse until now out of fear that her career would be affected” (Squires, 2021). Loss might come as a punishment for breaking in-group loyalty from those in positions of power within sport but can also come as a protective measure from the female athlete’s family, “at that time we did not have any collaboration with a sports psychologist, and of course I would never talk to my parents because they would have stopped me from sailing” (Squires, 2021). In both cases, the athlete has to negotiate between losing her sport dream and breaking free from abuse, and those who speak up feel as if they can help, “I am trying to help where it is needed so that other women can have dreams” (Kottis, 2021).

While an in-group expects loyalty and solidarity from its members, there are times when staying silent as an act or loyalty to the sport may oppose to solidarity with group members that one also wants to protect. Remaining silent and not letting the public know about the abuse and abuser contradicts solidarity toward the children and youth members of the group who cannot be protect if information on the abuse and abuser were not shared publicly. Majority of the women who came forward, found the strength to speak up in order to protect the future generation. As another athlete said, “she decided to speak up to ‘preserve the health and safety’ of young athletes” (Public Radio International, 2021). The women saw more weight in protecting future generations from abuse than in wining gold, “To bring back a gold medal to your country …was great but it did not last long. ‘This change, I hope, will last and protect future generations’” (Kottis, 2021). These words also reveal how well-ingrained are collectivist values of family and the protection of its members at all costs. The women found strength to speak up in the thought of saving younger children and not of saving themselves, after all they are women who were primed as second-rate citizens of Greece (Lolis, 2020; Tsiganou, 2021).

The theme of *preserving power* was grounded on sexual abuse being about the exploitation of power relations and not about sex, and the corrupted management practices that run Greek sport as ways for supremacy, power, control, and authority to stay with those who possess them. In the data we reviewed, athletes shared how their naked bodies were exploited by persons of authority, “Mania Bikof, a retired water polo player said she had been forced to strip to the waist so a doctor could examine a shoulder injury, and the former swimming champion Rabea Iatridou, said she had been groped by a medic” (Kitsantonis, 2021). According to social theorists controlling a person’s body is one of the most effective ways to obtain control of the individual (Foucault, 1978, 1979; Bourdieu, 1986; Giddens, 1994), while in the majority of cases sexual harassment and abuse, like the ones we find within the Greek in-group of sport, are not about sex but control in asymmetrical power relations (Fasting and Brackenridge, 2009). The data also revealed that some Greek female athlete-survivors have come to understand that sexualized acts and/or sex are a means of exerting power in close relations where power and trust are abused (Chroni, 2013b), “‘My personal experience is not an individual issue that affects only me… It’s part of a wider and chronic problem regarding abuse of power in general but also particularly with the current [federation] leadership’” (Angelopoulou and Tsafos, 2021). And while persons of power can misuse it, athletes are expected to stay quiet (loyal to the group), “Male athletes have spoken out too, including Nikos Kaklamanakis, another sailing champion, who said that sailing federation officials threatened young athletes to stay quiet about alleged abuses” (Kitsantonis, 2021).

Greece ranks high on corruption and is often constructed as an untrustworthy state that needs help from European institutions to fight corruption and bribery through projects and awareness raising campaigns (Organisation for Economic Co-operation and Development, n.d.). Corrupted management is also present in Greek sport and measures were undertaken by the ministry of sport aiming to improve sport federations and clubs’ governance (General Secretariat of Sport, 2021). Within our data, corrupted management was constructed as a framework that sustained abuse instead of safeguarding athletes,

*Bekatorou’s fellow-athlete Nikos Kaklamanakis, also a decorated sailing champion, spoke in parliamentary committee of gross financial misconduct in the sailing federation. “If we’re asking you to tear down walls today, it is to tear down walls of corruption, abuse of power and neglect,” Kaklamanakis said. The sailing federation sued him for defamation. Bekatorou was called as a witness. During her deposition last November, she denounced her rape [from a federation official] for the first time. (Psaropoulos, 2021)*

As elaborated in the data, “Bekatorou… was among several top sailors who last year publicly accused the federation of mismanagement and creating a ‘toxic’ environment to bully and control athletes” (Vassilopoulos, 2021). In a corrupted sport environment, values, processes, and rules are most likely not in place and the mismanagement reported above allows for exerting control over athletes in any way possible even with violence.

In closing, it appears that in these failed negotiations with power, the collective and individual ends meet once again but this time on challenging grounds requiring them to negotiate between one’s well-being, integrity and social and sporting cultural aspects or how power is retained by authority structures and persons. The theme of failed negotiations with power, suggests that wrongdoings are not confronted, and that power remains on the side that it was. A key message here is that authority structures and persons in Greece remain protected at all costs.

### Concluding Thoughts

In the present study, we engaged with cultural praxis (Ryba and Wright, 2005, 2010; Chroni and Kavoura, 2020) to explore how the cultural context in Greece operates as an underlying factor in priming athletes for harassment and abuse and in oppressing them from speaking up, asking for help, and advocating for safer sport. Specifically, our thematic analysis of media data revealed two overarching trends in relation to how the Greek sport context conditions athletes, namely, *keeping the home intact* and *failed negotiations with power*. Keeping the home intact is carried out through outdated and untrustworthy practices that teach women athletes blindfolded discipline, disempower them and discourage victim-survivors to speak up, while also failing to provide the sport context with improvements that can support women athletes in contesting maltreatment and exploitative behaviors, coming forward, and finding a pathway to healing. Failed negotiations with power involve individual athletes having to constantly negotiate with those who preserve the power (in this case, corrupted male federation officials and other men who abuse their positions of power), as well as with national- and sport-culture glitches. The decision whether to speak up about abuse and save future generation yet ruin their relationships with coaches and federation officials and therefore their athletic career, or to stay silent and continue the pursuit of their Olympic dream, is a difficult one to make. Their upbringing inside collectivism and the loyalty to family and its members (e.g., children) that is instilled in them appear to shape the drive to break their silence even without a safety net of proper reporting procedures.

The themes identified in this paper can be viewed as expressions of how collectivism and patriarchy are performed in everyday life (also outside the realm of sport). The outdated and abusive coaching, training practices reproduce and retain the supremacy of men in power. The untrustworthy, outdated system and practices preserve gender hierarchy and inequalities against women who are targeted by violence more than men and perpetuate traditional stereotypes through individual interpretations of laws performed by law enforcers. Promises on policy changes that do not happen, is an indirect way to keep things as they are and retain power and authority of men. The negotiations that athletes-survivors face are indications of a collectivist society that expects to remain “clean” of wrongdoings by hiding these (culture of silence) and a sport community that glorifies everything about men, male prowess, and male dominance. Lastly, the theme of preserving power is the ultimate illustration about how male authority is retained through wrongdoings, such as violence and corruption.

While this study is based on a rather small number of national and international articles, that concerned nine cases of sexual violence in Greek sport published between January 2021 and January 2022, it is crucial to note here the absence of research on sexual violence in Greece. The fact that since Chroni and Fasting (2009) collected data on female athletes’ experiences with sexual harassment no other study has been conducted may be a signal of how incidents of violence remain a taboo that Greeks do not expose and the challenge of conducting research on such sensitive topic. We could probably have identified more cases, from more sporting contexts, if we had broadened our search terms and/or the timeline but reaching saturation in codes found in the data assured us that we could go ahead with writing this manuscript. We firmly believe that our study provides important information about the cultural elements that delayed the arrival of the #metoo and continues to hold back change and progress with regard to combating sexual violence in Greek sport as well as other forms of violence, including physical and emotional violence. Our findings have important implications for understanding what lays behind the occurrence of sexual violence in the Greek sport context and victims-survivors being hesitant to come forward. In light of these findings, and if we want to safeguard athletes against sexual violence, such socio-cultural elements need to be brought to light and addressed within any policy and measures. For safeguarding and safe sport to succeed, the development and implementation of policy and measures must be grounded in the context in which violence occurs. To achieve this, cultural praxis could be used as a heuristic, and as a “how to” of combing localized research, with critical theory and actual praxis in the field (Chroni and Kavoura, 2020). As Fisher and Anders (2019) argued, tackling sexual violence in sport requires us to re-center efforts in exploring how power dynamics and hierarchies are integrated in specific cultural contexts, and in dismantling the cultural ideologies and politics that might be linked to exploitative behaviors, and in our case, also to resistance to change.

The findings presented here are in congruence with previous scholarly work (Lolis, 2020; Tsiganou, 2021) that links sexual abuse with persisting manifestations of patriarchy and collectivism, as these can be traced in the Greek climate of silence, the under-reporting, the defying of victim credibility, or the victim blaming and shaming. Socio-cultural nuances that lay behind the themes of keeping the home intact and failed negotiations with power, condition all involved for wrongful use of power, unreported misconduct of sexual nature and provide foundation to the walls of silence that surround sexual violence in Greek sport. Under current circumstances, it seems as if change is a threat to all involved, yet for different reasons. For the coaches, sport officials, stakeholders, and the state system, change would require them to relinquish male powers and authority, find new meaning of what it means to be and do as a man, and allow women to be seen as counterparts. For the female athlete-survivors, it would require them to prioritize the self and their self-care and let go of the in-group loyalty and subordination they learned to be and show since infancy.

## Data Availability Statement

The data generated and analyzed during this study are included in this published article/supplementary material, further inquiries can be directed to the corresponding author.

## Author Contributions

SC initiated and led the study, wrote the manuscript, identified the focal area, reviewed the literature, was a critical friend in data analysis, and shaped data interpretation and the development of findings. AK collected and analyzed the data as a specialist on cultural praxis, was instrumental in the development of findings, and wrote the manuscript. Both authors contributed to the article and approved the submitted version.

## Conflict of Interest

The authors declare that the research was conducted in the absence of any commercial or financial relationships that could be construed as a potential conflict of interest.

## Publisher’s Note

All claims expressed in this article are solely those of the authors and do not necessarily represent those of their affiliated organizations, or those of the publisher, the editors and the reviewers. Any product that may be evaluated in this article, or claim that may be made by its manufacturer, is not guaranteed or endorsed by the publisher.
